# Editorial: Cognitive Multitasking – Towards Augmented Intelligence

**DOI:** 10.3389/fnins.2021.619090

**Published:** 2021-01-22

**Authors:** Liang Feng, Yew Soon Ong, Huajin Tang, Will Neil Browne

**Affiliations:** ^1^College of Computer Science, Chongqing University, Chongqing, China; ^2^School of Computer Science and Engineering, Nanyang Technological University, Singapore, Singapore; ^3^College of Computer Science and Technology, Zhejiang University, Hangzhou, China; ^4^School of Engineering and Computer Science, Victoria University of Wellington, Wellington, New Zealand

**Keywords:** cognitive multitasking, multitask learning, transfer optimization, evolutionary multitasking, guest editorial

The original inspiration of artificial intelligence (AI) was to build autonomous systems that were capable of demonstrating humanlike behaviors. However, modern AI systems have begun to far exceed humanly achievable performance levels in areas such as image processing, complex optimization, and unmanned systems, due to the present-day data deluge, accompanied by subtle algorithmic enhancements in machine learning algorithms. This is occurring across a variety of domains, where prominent examples include IBM Watson winning Jeopardy! and Google DeepMind's AlphaGo beating the world's leading Go player. However, the AI future need not be limited to a human imitating standpoint. Instead, it may be more beneficial to build AI systems that are able to excel at that which humans have not evolved to do or to even consider. Humans have not evolved to process multiple distinct situations within short timespans (i.e., in the order of a few seconds) – as interleaving more than one task usually entails a considerable switching cost during which the brain must readjust from one task to the other.

Machines, on the other hand, are largely free from any such switching bottlenecks. Thus, machines can move more fluidly between tasks. Furthermore, when related tasks are bundled together, it may also be possible to seamlessly transfer or share the learned knowledge among them. As a result, while an AI attempts to solve some complex task, several other simpler ones may be unconsciously solved. Moreover, knowledge learned unconsciously in one task may be harnessed for intentional use in another application.

This special issue aims to explore deeply the issues faced in cognitive multitasking. Emphasis is placed on computational models and algorithms, as well as new hardware advances, that shall enable machines to be developed as consummate multitask problem-solvers. Following a rigorous peer review process, 11 papers have been accepted to be included in the special issue.

The first paper, “Multi-Task Learning Based Network Embedding” by Wang et al. presents a multi-task learning-based network embedding approach for network representation learning. The first task is designed to preserve the high-order proximity between pairwise nodes, while the second task is to preserve the low-order proximity in the one-hop area of each node. Comprehensive empirical studies on multi-label classification, link prediction, and visualization in five real-world networks, including social network, citation network, and language network, have been conducted to evaluate the performance of the proposed method over existing state-of-the-art approaches.

In the second paper entitled “High Cognitive Flexibility Learners Perform Better in Probabilistic Rule Learning,” Feng et al. analyze how cognitive flexibility of human being, as assessed by the number-letter task, is associated with the learning process of a probabilistic rule task. This paper concludes that further research should be conducted to explore the internal process of learning differences between high and low flexibility learners by using other technologies across multiple modes.

To improve the convergence speed, a two-level transfer learning method has been proposed by Ma et al. in their paper “A Two-Level Transfer Learning Algorithm for Evolutionary Multitasking.” The proposed method intends to use the correlation and similarity among the paired tasks to improve the efficiency and effectiveness of a multifactorial evolutionary algorithm.

The forth paper, “A Preliminary Study of Knowledge Transfer in Multi-Classification Using Gene Expression Programming” by Wei and Zhong. embarks a preliminary study on evolutionary multitasking optimization with gene expression programming for multi-classification. Experimental studies on 10 high-dimensional datasets show that knowledge transfer among separate binary classifiers under the proposed multitasking method can enhance multi-classification performance when compared to existing approaches.

To learn good representations of node in graphs or network, Xie et al. proposed a multi-task representation learning architecture coupled with the task of supervised node classification for graph classification and an end-to-end multi-task network representation learning framework with multi-task loss function for network embedding, in “A Multi-Task Representation Learning Architecture for Enhanced Graph Classification” and “Multi-Task Network Representation Learning,” respectively.

In the seventh paper entitled “Droplet-Transmitted Infection Risk Ranking Based on Close Proximity Interaction,” to identify people who are potentially-infected by droplet-transmitted diseases, Guo et al. present a multi-tasking framework to model the principle of Close Proximity Interaction and thus infer the infection risk of individuals. Experimental studies in different scenarios, including indoor office, bus station and bus compartment, hospital, show that the proposed method can achieve consistent results when compared to manual analysis very efficiently.

The eighth paper, “A Privacy-Preserving Multi-Task Learning Framework for Face Detection, Landmark Localization, Pose Estimation, and Gender Recognition” by Zhang et al. introduce a privacy-preserving multi-task learning approach to address the privacy issue existing in the training data for face processing tasks. The proposed method utilizes the differential private stochastic gradient descent algorithm to optimize the end-to-end multi-task model and weighs the loss functions of multiple tasks to improve learning efficiency and prediction accuracy.

To improve the performance of multi-task optimization, Xu et al. present new transfer sparks in fireworks algorithm for multitasking. For each task to be optimized, transfer sparks are generated with adaptive length and promising direction vector to transfer useful genetic information between different tasks while the optimization progresses online. The efficacy of the proposed method has been validated on the multi-task optimization benchmarks against existing state-of-the-art evolutionary multitasking approaches.

In the 10th paper entitled “Electroencephalographic Workload Indicators During Teleoperation of an Unmanned Aerial Vehicle Shepherding a Swarm of Unmanned Ground Vehicles in Contested Environments,” Rojas et al. try to identify the electroencephalographic (EEG) indicators that can be used for the objective assessment of cognitive workload in a multitasking setting and as a foundational step toward a human-autonomy augmented cognition system.

Last but not the least, Howard et al. in their paper “BrainOS: A Novel Artificial Brain-Alike Automatic Machine Learning Framework,” explores some of the principles of the brain that seem to be responsible for its autonomous, problem-adaptive nature. The presented BrainOS is an automatic approach for selecting the appropriate model based on three factors, which are (a) input at hand, (b) prior experience, which is a history of results of prior problem solving attempts), and (c) world knowledge that represented in the symbolic way and used as a means to explain its approach. Preliminary studies of BrainOS show that it can deal with complex problems, such as natural language processing.

As can be observed, in the 11 accepted papers, the second and tenth paper focus on psychology and neuroscience in cognitive multitasking, while the other papers concentrate on the multi-task optimization and learning algorithm designs. The human brain possesses the most remarkable ability to perform multiple tasks with apparent simultaneity, and leverages the experiences in solving one task to help the decision making in another task. These accepted papers have first illustrated the explorations of intelligent systems and algorithms that mimic beyond the human brain in efficient multitasking. Secondly, it can also be observed in these papers that the rapid increase in the variety, volume and complexity of real-world problems, the opportunity, tendency, and (even) the need to multitask is unprecedented.

The guest editors would like to thank all the authors who submitted their work to the special issue, and all reviewers for their hard work in completing timely and constructive reviews. Special thanks also go to the Editor-in-Chiefs and members of the editorial team for their support during the editing process of this Special Issue.

## Author Contributions

LF was the corresponding guest editor. All the authors are the guest editors of this special issue.

## Conflict of Interest

The authors declare that the research was conducted in the absence of any commercial or financial relationships that could be construed as a potential conflict of interest.

